# Underwater Acoustic Target Detection Using a Miniaturized MEMS Hydrophone Array

**DOI:** 10.3390/mi17040468

**Published:** 2026-04-12

**Authors:** Xiao Chen, Ying Zhang

**Affiliations:** 1 Information Science and Technology College, Dalian Maritime University, Dalian 116026, China; 2 Department of Electronic Engineering, Ocean University of China, Qingdao 266100, China; zhying_note@163.com

**Keywords:** MEMS acoustic pressure hydrophone, miniaturized hydrophone array, phase error, target detection, differential beamforming

## Abstract

Sonar is a fundamental tool for underwater target detection. However, conventional detection systems often suffer from poor sensor consistency and high fabrication costs. More critically, for low-frequency operation, the required array aperture becomes prohibitively large, limiting their deployment on small, mobile underwater platforms. To address the demand for compact, high-performance sensing solutions, this paper presents a miniaturized Micro-electromechanical Systems (MEMS) hydrophone array designed for underwater target detection. The array consists of six elements with a spacing of 0.25 m. Each element is approximately 22 mm in diameter and encapsulated in polyurethane via a casting and curing process. The core sensing element, a MEMS acoustic pressure hydrophone, exhibits a sensitivity of −177.2 ± 1.5 dB (re: 1 V/µPa) across the 20 Hz to 4 kHz frequency range and a noise resolution of approximately 59.5 dB (re: 1 µPa/√Hz) at 1 kHz. A key challenge in array-based detection is the phase mismatch among acquisition channels, which degrades algorithm performance. To mitigate this, we propose a phase self-correction method based on interleaved ADC acquisition control, enabling synchronous multi-channel sampling and effectively eliminating system-level phase errors. Furthermore, to overcome the inherent aperture limitations of conventional beamforming (CBF) applied to a miniaturized array, a differential beamforming (DBF) algorithm is adopted. This approach is less frequency-dependent and can approximate a frequency-invariant beam pattern, making it well-suited for miniaturized arrays. Simulation results confirm the theoretical validity of the DBF algorithm for the proposed MEMS hydrophone array. Sea trial data further demonstrate that this method achieves higher target detection accuracy compared to CBF techniques.

## 1. Introduction

Underwater detection relies primarily on sonar, a technology that uses sound waves for navigation, communication, and target identification on vessels and submarines. Central to sonar functionality is the hydrophone, an acoustic sensor that receives sound waves and converts them into electrical signals. By capturing underwater acoustics signals, hydrophones enable critical applications in marine exploration, environmental monitoring, and ocean development [1,2,3,4].

Traditional hydrophones are often limited by poor consistency and high manufacturing costs. Moreover, when applied to low-frequency detection, the required array aperture becomes prohibitively large, making them unsuitable for deployment on mobile underwater platforms. In recent years, MEMS technology has emerged as a transformative advancement in sensor development. Integrating MEMS with sonar technology enables the realization of compact, batch-fabricated, low-cost detection systems, offering significant potential for underwater acoustic applications. Several MEMS hydrophone designs have been reported in the literature. Sung et al. proposed a MEMS piezoelectric hydrophone based on a field-effect transistor gate structure, achieving a sensitivity of −191.5 dB over a 50 Hz to 1 kHz bandwidth [5]. Xu et al. developed an aluminum nitride (AlN)-based MEMS piezoelectric hydrophone for low-frequency operation (10–100 Hz), with a chip area of 2 mm × 2 mm and a sound pressure sensitivity of −182.5 dB [6]. Yang et al. designed a high-sensitivity, low-noise AlN-based MEMS hydrophone with a chip size of 3.5 mm × 3.5 mm, an operating bandwidth of 100–1600 Hz, a sensitivity of −178 dB, and a noise resolution of 52.6 dB at 100 Hz, suitable for marine ambient noise monitoring [7]. MEMS hydrophones are currently used mainly for communication and positioning, with limited application in target detection. This paper addresses this gap by designing a miniaturized MEMS hydrophone array that capitalizes on the technology’s inherent advantages—small size, light weight, good consistency, and ease of integration—for underwater target detection.

Phase errors in the output signals of a MEMS hydrophone array can arise from several factors, including inconsistencies in the phase response of individual array elements, variations in the acquisition circuitry, and installation misalignments. These phase deviations distort the array’s directivity pattern and compromise the accuracy of underwater target detection, necessitating effective phase error correction. Existing correction methods are generally categorized as either passive or active. Passive correction methods utilize environmental noise or the target signal itself, employing algorithms such as genetic algorithms or particle swarm optimization to estimate phase errors [8,9]. While these methods exhibit strong environmental adaptability, they often suffer from high computational complexity, and the uncertain nature of ambient noise can compromise the convergence of parameter estimation. In contrast, active correction methods rely on an external auxiliary source and are typically combined with algorithms such as multiple signal classification or weighted least squares to calibrate phase errors in a controlled environment [10,11,12]. This paper proposes a Field Programmable Gate Array (FPGA) based phase self-correction method using interleaved Analog-to-Digital Converter (ADC) acquisition. Unlike conventional active correction methods that perform feedback compensation in the digital domain, the proposed method performs feedforward phase compensation directly in the hardware domain. This approach corrects phase deviations across the entire MEMS underwater acoustic array acquisition system, thereby mitigating the accuracy degradation in underwater target detection caused by such phase errors.

Underwater target detection using a MEMS hydrophone array critically depends on the design of signal processing algorithms. In CBF, the spatial Nyquist sampling theorem must be satisfied to ensure effective processing. Specifically, the inter-element spacing should not exceed half the wavelength of the incident signal [13,14,15]. Exceeding this limit leads to beam broadening and increased sidelobe levels, which severely degrades detection performance [16]. Furthermore, in engineering applications, the physical dimensions of MEMS hydrophone arrays are often constrained by platform volume and installation conditions, thereby limiting their effective aperture [17]. Under such compact configurations, CBF algorithms typically yield low directional gain, making them inadequate for reliable low-frequency signal detection. To address these limitations, this paper adopts a DBF algorithm. Leveraging its unique signal processing mechanism, the proposed approach overcomes the strict element-spacing constraints imposed by the Nyquist criterion, enabling stable target detection with a miniaturized MEMS hydrophone array. This work thus provides a feasible technical pathway for array-based detection on small underwater platforms.

Based on the above analysis, this paper presents the integrated design of a miniaturized MEMS hydrophone array, leveraging the inherent advantages of MEMS hydrophones—namely, small size, good consistency, and ease of integration. To meet the requirements of low-frequency underwater target detection using miniaturized MEMS hydrophone arrays, differential beamforming algorithms are investigated. To address the high sensitivity of differential beamforming to the phase consistency among array channels, an FPGA-based phase self-correction method using interleaved ADC acquisition is designed. Finally, the underwater target detection system incorporating this small-sized MEMS hydrophone array was tested in a real sea environment.

## 2. Materials and Methods

### 2.1. MEMS Piezoelectric Hydrophone

The MEMS piezoelectric sensing chip detects acoustic signals through the direct piezoelectric effect. When the piezoelectric material deforms under external force, the resulting change in its electric dipole moment induces polarization, generating bound charges of opposite polarity on the film surface [18]. The accumulated charge is proportional to the applied mechanical stress, i.e., the intensity of the incident sound wave.

**Figure 1:**
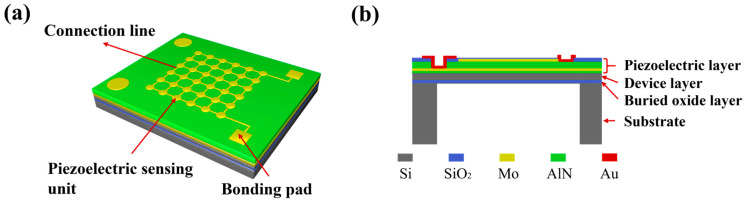
(**a**) 3D schematic of the MEMS sensing chip. (**b**) Cross-sectional view of a piezoelectric sensing unit.

The MEMS sensing chip integrates a 6 × 6 array of piezoelectric sensing units. As illustrated in Figure 1a, these units are electrically connected in parallel via their metal electrodes. The cross-sectional structure of a single unit is shown in Figure 1b, comprising a piezoelectric layer, a device layer, a buried oxide layer, and a silicon substrate. The piezoelectric layer consists of a piezoelectric film topped with a metal electrode, which collects the charge generated on the film surface. The device layer and buried oxide layer are formed from device silicon and silicon dioxide (SiO_2_), respectively. A back cavity is created by deep reactive-ion etching of the handle layer from the backside of the silicon-on-insulator (SOI) substrate, forming a suspended membrane structure. When excited by an incident acoustic pressure wave, this membrane undergoes strain-induced bending, causing the piezoelectric film to experience alternating compression and tension. Consequently, charges of corresponding polarity are induced on the film surface. These charges are collected by the metal electrodes and transmitted to the subsequent signal conditioning circuit, thereby producing an electrical output proportional to the acoustic signal.

Piezoelectric film serves as the sensitive element of MEMS hydrophones, and its properties directly determine the device’s sensitivity [19]. Specifically, hydrophone sensitivity is proportional to the figure of merit (FOM) of the piezoelectric material, defined as the ratio of its piezoelectric constant to its dielectric constant. A higher FOM value corresponds to higher achievable sensitivity. Commonly used piezoelectric thin-film materials include lead zirconate titanate (PZT), zinc oxide (ZnO), and aluminum nitride (AlN). Their key properties are summarized in Table 1. Although AlN exhibits a lower piezoelectric coefficient than PZT, its significantly lower dielectric constant results in a higher FOM. ZnO offers high FOM but suffers from poor chemical stability, limited corrosion resistance, and incompatibility with CMOS processes, restricting its practical application. Based on this comparative analysis, AlN was selected as the piezoelectric material for the MEMS hydrophone in this work.

**Figure 2:**
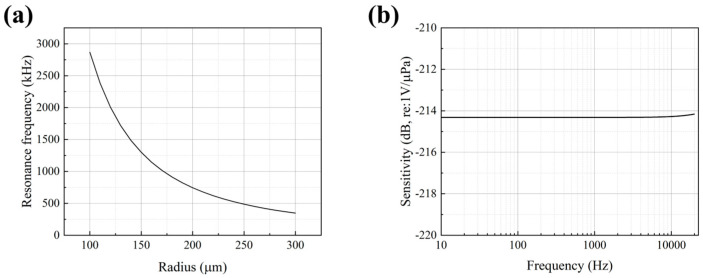
(**a**) Relationship between resonant frequency and diaphragm radius for the MEMS sensing chip. (**b**) Simulated sensitivity curve of the MEMS sensing chip.

A finite element model of the MEMS piezoelectric sensing chip was established in COMSOL Multiphysics 6.3 to perform modal analysis. The key geometric parameters of the finite element model are listed in Table 2. Using the modal analysis module in COMSOL, the relationship between the vibrating diaphragm radius and the resonant frequency can be determined. This allows the selection of a suitable diaphragm radius to achieve the desired resonant frequency. Figure 2a plots the resonant frequency of the MEMS piezoelectric sensing chip as a function of the diaphragm radius. The figure reveals that the resonant frequency is inversely proportional to the diaphragm radius. Considering fabrication constraints, a diaphragm radius of 250 μm was selected. Figure 2b shows the simulated sensitivity of the MEMS sensing chip, which is approximately −214.2 dB (re: 1 V/μPa).

**Figure 3:**
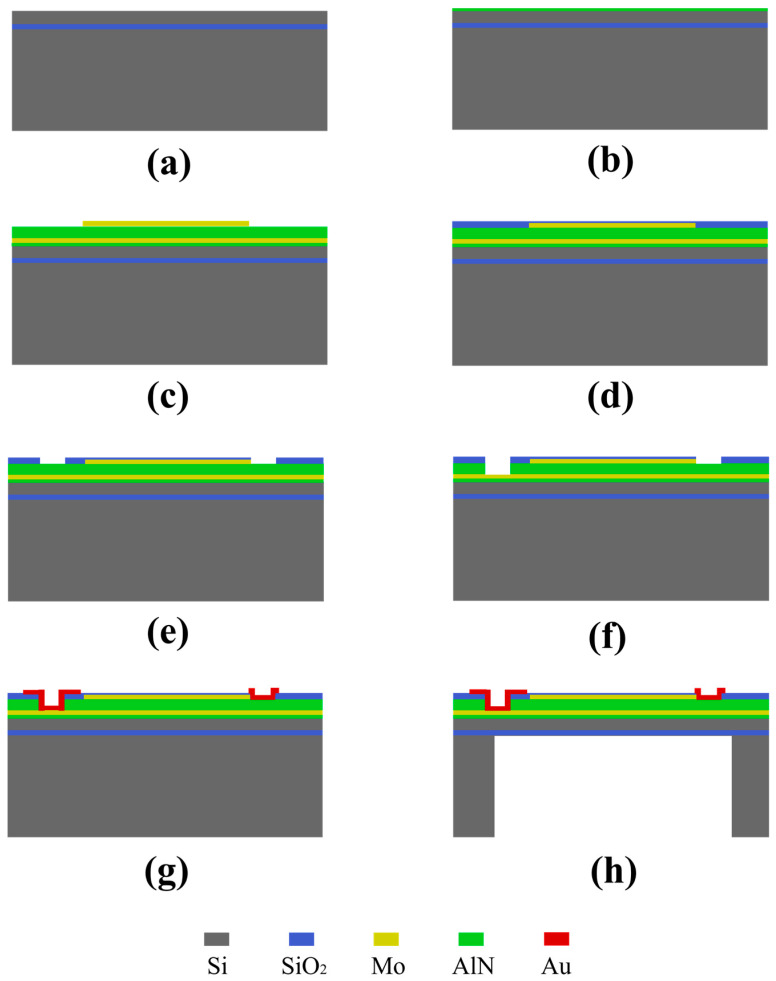
Fabrication process flow of the MEMS sensing chip. (**a**) Substrate cleaning and preparation. (**b**) AlN seed layer deposition. (**c**) Magnetron sputtering of Mo/AlN/Mo stack. (**d**) SiO_2_ protective layer deposition. (**e**) Patterning of SiO_2_ protective layer. (**f**) Wet etching of AlN. (**g**) Metal lift-off for Au bonding pads. (**h**) Backside DRIE for cavity release.

The MEMS sensing chip was fabricated using bulk silicon micromachining technology, with the main process flow illustrated in Figure 3. A SOI wafer with a 5 µm device layer, a 1 µm buried oxide layer, and a 400 µm handle layer was used as the starting substrate. After standard cleaning, a multilayer stack of AlN (20 nm)/Mo (300 nm)/AlN (1.8 µm)/Mo (300 nm) was deposited by magnetron sputtering. The initial 20 nm AlN layer serves as an adhesion promoter. The top Mo electrode was patterned using photolithography and etching according to the designed electrode geometry. A 0.5 µm SiO_2_ protective layer was then deposited by chemical vapor deposition (CVD) to shield the AlN regions not requiring patterning from subsequent processing steps. This protective layer was selectively etched to expose areas for further AlN etching and to open contact windows for the metal electrodes. Wet etching of AlN was performed to expose the bottom electrode. Subsequently, a 250 nm Au layer was deposited by magnetron sputtering and patterned via lift-off to form bonding pads for wire interconnection. Finally, deep reactive-ion etching (DRIE) was performed from the backside of the SOI wafer. Exploiting the etch selectivity between Si and SiO_2_, the silicon handle layer was etched down to the buried oxide layer, thereby releasing the vibrating diaphragm.

**Figure 4:**
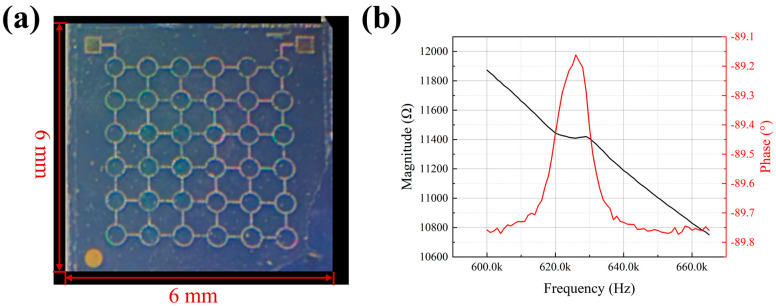
MEMS sensing chip characterization. (**a**) Optical image of the 6 mm × 6 mm chip. (**b**) Measured impedance and phase curves as a function of frequency.

The fabricated MEMS sensing chip is shown in Figure 4a, with dimensions of approximately 6 mm × 6 mm. Its impedance characteristics were measured using a 4200-SCS semiconductor parameter analyzer (Keithley Instruments, Inc., Cleveland, OH, USA). An AC voltage signal with an amplitude of 1 V was applied to the output terminals of the chip, and the frequency was swept to obtain the impedance response across different frequencies. The resulting impedance curve is presented in Figure 4b. As can be observed, the resonant frequency of the chip in air is approximately 622 kHz. The simulated resonant frequency of the chip is approximately 500 kHz. This notable discrepancy can be primarily attributed to dimensional errors and residual stresses inherent in MEMS fabrication, as well as resonance of the testing fixture, which also compromises measurement accuracy.

The MEMS piezoelectric hydrophone integrates the sensing chip and its preamplifier circuit within a single package, thereby minimizing signal transmission distance and reducing susceptibility to interference during signal transfer. To minimize the device’s self-noise, the preamplifier is implemented as a low-noise, two-stage amplifier circuit based on junction field-effect transistors, with an amplification factor of 40 dB [20]. For underwater operation, an additional encapsulation matching layer is required to ensure efficient acoustic transmission, waterproofing, and corrosion resistance. Polyurethane was selected as the encapsulation material due to its acoustic impedance closely matching that of water, low sound attenuation coefficient, and excellent corrosion resistance.

**Figure 5:**
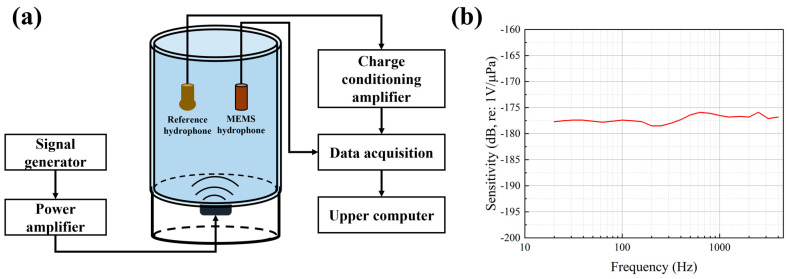
Sensitivity calibration of the MEMS piezoelectric hydrophone. (**a**) Schematic of the standing wave tube calibration setup. (**b**) Measured sensitivity curve.

The sensitivity of the MEMS piezoelectric hydrophone was calibrated in two different acoustic environments: a standing wave tube for the 20 Hz–1 kHz frequency band, and an anechoic tank for the 1–4 kHz band. A comparison method was employed using a B&K 8105 hydrophone (Brüel & Kjær Sound & Vibration Measurement A/S, Nærum, Denmark) as the reference. The calibration results are presented in Figure 5. As shown, the hydrophone exhibits a sensitivity of −177.2 dB (re: 1 V/µPa) with a fluctuation within ±1.5 dB across the entire 20 Hz to 4 kHz range, demonstrating a flat frequency response. A discrepancy of approximately 3 dB exists between the measured sensitivity and the simulated value (−174.2 dB re 1 V/μPa after preamplifier inclusion). This difference is primarily attributable to manufacturing inaccuracies and packaging-related factors.

**Figure 6:**
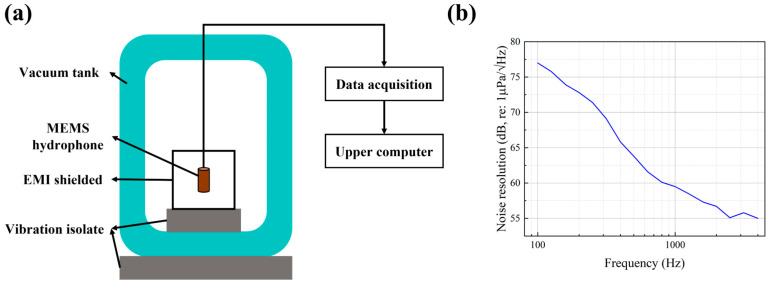
Noise resolution measurement of the MEMS piezoelectric hydrophone. (**a**) Schematic of the test setup. (**b**) Measured noise resolution curve.

The self-noise performance of the MEMS piezoelectric hydrophone was evaluated in a vacuum tank. To isolate external acoustic interference, both the inner and outer walls of the tank were lined with sound-absorbing foam, and the chamber was evacuated. The measured noise resolution is shown in Figure 6. The results indicate that the hydrophone exhibits a low self-noise level, with a noise resolution of approximately 59.5 dB (re: 1 µPa/√Hz) at 1 kHz.

### 2.2. MEMS Hydrophone Array

**Figure 7:**
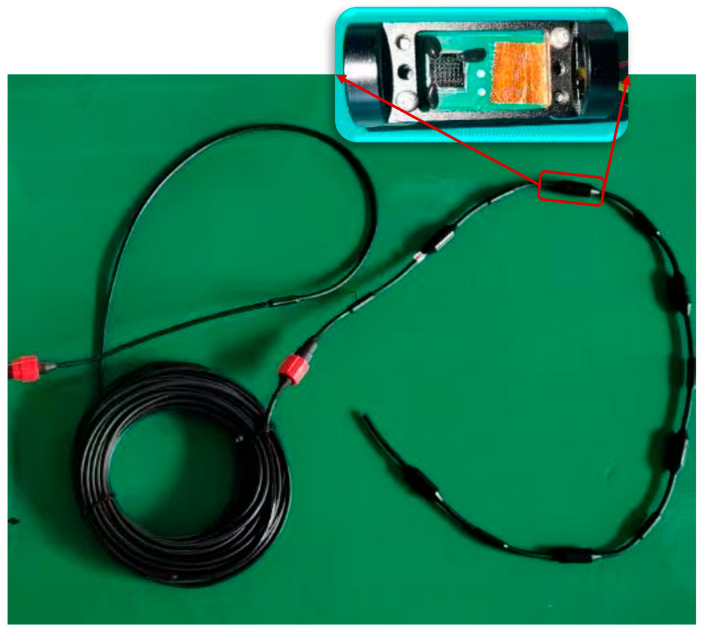
Photograph of the six-element MEMS hydrophone linear array.

A single MEMS piezoelectric hydrophone can only capture pressure information. To enable target detection, a MEMS hydrophone array was designed to exploit the array’s directional properties and the time delay differences between elements. The array consists of six MEMS acoustic pressure hydrophones, each sharing a common power supply while their output signals are routed independently. The hydrophones were assembled and secured using a custom packaging mold, and the entire array was encapsulated in polyurethane via a molding and vulcanization process. The fabricated array is shown in Figure 7. It features six elements with an inter-element spacing of 0.25 m, each approximately 22 mm in diameter.

### 2.3. Phase Correction of the MEMS Hydrophone Array Acquisition System

**Figure 8:**
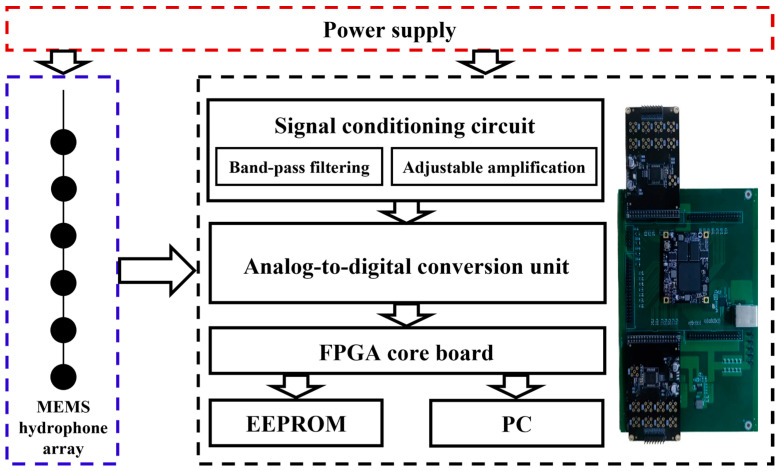
Schematic diagram of the MEMS hydrophone array acquisition system.

The MEMS hydrophone array acquisition system comprises three main components: a signal conditioning circuit, an FPGA control circuit, and a power supply unit. As illustrated in Figure 8, the signal conditioning circuit performs band-pass filtering and amplification on the signals received by the hydrophone array. After filtering, the signal is amplified with adjustable gain to ensure its amplitude falls within the analog input range of the subsequent ADC stage. Upon receiving a command from the FPGA, multiple ADCs simultaneously sample the conditioned signals and transmit the digitized data in parallel to the FPGA. The FPGA performs subsequent processing, stores the results in an EEPROM, and forwards them to the host computer.

The phase consistency of the acquisition channels in a MEMS hydrophone array is a critical parameter for accurate underwater target detection. Phase deviations in the system primarily arise from two sources: element position errors and inconsistencies in device characteristics. Based on these factors, a theoretical model of phase deviation was established.

Assume the target signal is an ideal sinusoidal waveform *S*(*t*), given by

(1) $$S\left(t\right)=A\sin(2\pi ft+\theta_{0})$$

where *A*, *f*, and *θ*_0_ are the amplitude, frequency, and initial phase. With phase errors due to element mispositioning, the signal takes the form *S_n_*(*t*):

(2) $$S_{n}\left(t\right)=A_{n}\sin[2\pi ft+\theta_{0}+\Delta \theta {\left(f\right)}_{n}]$$

where $\Delta \theta {\left(f\right)}_{n}$ is the phase shift caused by element position disturbance, and *A_n_* (*n* = 1, 2, …, 6) is the amplitude received by the *n*-th array element. The acoustic signal, after being transduced by the MEMS sensing chip and conditioned through filtering and amplification, yields a processed signal given by:

(3) $$X_{n}\left(t\right)=B_{n}\sin[2\pi ft+\theta_{0}+\Delta \theta {\left(f\right)}_{n}+\Delta \varphi {\left(f\right)}_{n}]$$

where $\Delta \varphi {\left(f\right)}_{n}$ denotes the phase deviation due to device inconsistency, and *B_n_* is the amplitude after filtering and amplification.

The phase deviation between any two channels of the MEMS hydrophone array acquisition system is given by

(4) $$\Delta \phi {\left(f\right)}_{m}=\Delta \theta {\left(f\right)}_{q}+\Delta \varphi {\left(f\right)}_{q}$$

Among them,

(5) $$\begin{array}{cc} \Delta \theta {\left(f\right)}_{q}=\Delta \theta {\left(f\right)}_{i}+\Delta \theta {\left(f\right)}_{j} & i,j=1,2,3,4,5,6 \end{array}$$

(6) $$\begin{array}{cc} \Delta \varphi {\left(f\right)}_{q}=\Delta \varphi {\left(f\right)}_{i}+\Delta \varphi {\left(f\right)}_{j} & i,j=1,2,3,4,5,6 \end{array}$$

As evident from the above equation, the phase deviations $\Delta \theta {\left(f\right)}_{q}$ and $\Delta \varphi {\left(f\right)}_{q}$ vary with signal frequency. Therefore, wide-band phase correction is required.

**Figure 9:**
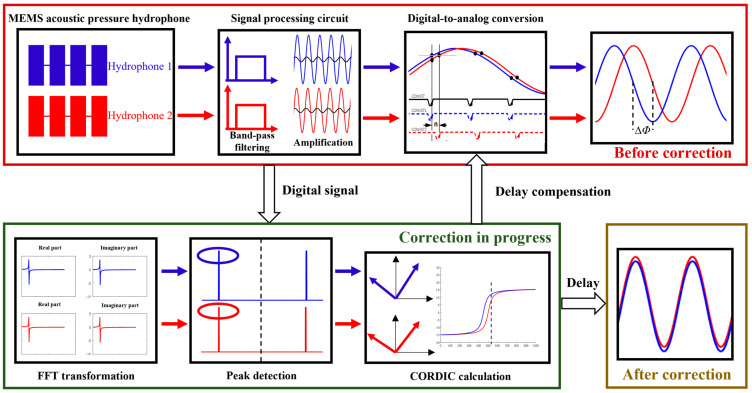
Schematic diagram of the phase self-correction method for the MEMS hydrophone array acquisition system.

To correct the phase deviations between channels in the MEMS hydrophone array acquisition system, an FPGA-based phase self-correction method was developed using interleaved ADC control. The correction procedure is illustrated in Figure 9. A standard sinusoidal signal is emitted from a sound source, and the initial phase of each channel’s acquired signal is obtained. The channel with the most advanced phase is selected as the reference, and the phase differences of the remaining channels are calculated relative to it. The phase difference calculation involves the following steps. First, the time-domain signals are transformed to the frequency domain via Fast Fourier Transform (FFT), and the real parts of the multi-channel signals are converted to the complex domain using an FFT IP core. Using the real and imaginary components obtained from the FFT, an arctangent calculation is performed with a CORDIC IP core to determine the phase difference between channels. Finally, the phase difference is converted into a corresponding delay, which is compensated by adjusting the timing of the ADC control signals. This delay compensation enables interleaved ADC acquisition, thereby achieving self-correction of channel phase deviations.

## 3. Signal Processing Results and Discussion

### 3.1. Beamforming Principle

Consider a far-field narrowband signal incident on a uniform linear array with *M* elements. The received signal can be expressed as

(7) X(t)=A(θ)g(t)+N(t)

where X(t) denotes the *M* × 1 array steering vector, N(t) is the *M* × 1 additive noise vector, g(t) is the *N* × 1 source signal vector, and A(θ) is the direction vectors corresponding to each source.

(8) $$A=[a_{1}(\omega_{0}),a_{2}(\omega_{0}),\ldots ,a_{N}(\omega_{0})]$$

(9) $$a_{i}(\omega_{0})=\left[\begin{array}{c} 1\\ e^{-j\omega_{0}\tau_{1i}}\\ \vdots \\ e^{-j\omega_{0}\tau_{Mi}} \end{array}\right],i=1,2,\ldots ,N$$

where $\omega_{0}=2\pi f=2\pi \frac{c}{\lambda }$, *c* is the speed of sound in water and *λ* is the wavelength, $\tau_{MN}$ denotes the time delay of the signal received by each array element.

**Figure 10:**
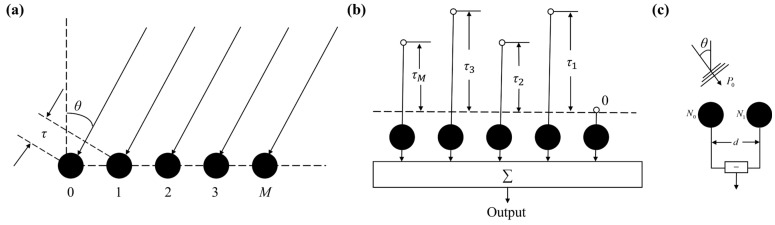
Linear array configuration and beamforming principle. (**a**) Wavefront arrival at a linear array. (**b**) Beamforming via delay compensation. (**c**) First-order differential array structure.

Beamforming technology enables directional signal enhancement and noise suppression by adjusting the phase of signals received by each element of a MEMS hydrophone array [21]. Because an incident wavefront reaches different array elements at different times, the received signals exhibit element-dependent phase differences. Beamforming compensates for these phase differences and may also adjust amplitudes, thereby aligning the signals to achieve constructive superposition in a target direction while canceling interferers from other directions. The underlying principle is illustrated in Figure 10.

After frequency-domain weighting of each array element’s signal, the beamformer output is obtained.

(10) $$X(\omega )=\sum_{m=1}^{M}{\bar{h}}_{m}(\omega )y_{m}(\omega )=H(\omega )Y(\omega )$$

where $H(\omega )={\left[h_{1}(\omega ),h_{2}(\omega ),\ldots ,h_{M}(\omega )\right]}^{T}$ denotes the weighting coefficient for each array element, $Y(\omega )=[y_{1}(\omega ),y_{2}(\omega ),\ldots ,y_{M}(\omega )]$ denotes the signal received by the array element.

CBF achieves optimal performance when the array elements are spaced at half-wavelength intervals. However, in practical engineering applications where the array aperture is constrained—such as with small-aperture arrays or size-limited platforms—the element spacing must be reduced. This reduction leads to a broadening of the main lobe width, resulting in degraded angular resolution. Conversely, if CBF is to be implemented with half-wavelength spacing under fixed aperture constraints, the number of array elements must be reduced. This reduction in element count decreases the array gain, thereby compromising the array’s target detection capability.

### 3.2. Differential Beamforming

The principle of DBF is to approximate the derivative of the acoustic pressure signal by taking the difference between the outputs of two adjacent array elements. This requires the element spacing to be much smaller than the half-wavelength of the signal, which implies a very small array aperture. This physical requirement is naturally satisfied by MEMS hydrophone arrays, making DBF well-suited to overcome the performance degradation of CBF at small element spacings [22]. Additionally, the algorithm is less sensitive to frequency variations and can produce a frequency-invariant beam pattern to a certain extent.

The structure of a first-order differential array composed of two omnidirectional hydrophones is shown in Figure 10c. Taking element *N*_0_ as the reference point, the inter-element spacing is denoted by *d*. For a far-field plane wave with amplitude *P*_0_ incident at an angle *θ*, the array output can be expressed as follows:

(11) $$Z(\omega ,\theta )=P_{0}(1-e^{-j\omega (\tau +d\cos\theta /c)})$$

where τ denotes the time delay of the array output signal. When the inter-element spacing is much smaller than the signal wavelength, the associated phase variation can be neglected, and the Maclaurin expansion yields

(12) $$Z(\omega )\approx P_{0}(\tau +d\cos\theta /c).$$

The response of the first-order differential array can be simplified by defining the following variables:

(13) $$a_{0}=\alpha_{1}=\frac{\tau }{\tau +d/c}$$

(14) $$a_{1}=1-\alpha_{1}=\frac{d/c}{\tau +d/c}.$$

Consequently, the final expression can be written as

(15) $$a_{0}+a_{1}=1.$$

The normalized array response is given by

(16) $$\bar{Z}(\theta )=a_{0}+a_{1}\cos\theta .$$

It can be observed that the array response attains its maximum value of unity at end-fire (0°), indicating that the main lobe is steered toward this direction, where the array exhibits the highest sensitivity. Following the same principle, the beam pattern representation for higher-order differential arrays can be formulated accordingly.

(17) $$B_{D,N}=\sum_{n=0}^{N}a_{n}\cos^{n}\theta$$

where $a_{n},n=0,1,\ldots ,N$ denotes the order of the difference array. Accordingly, the normalized responses for the first-order and second-order differential arrays can be expressed as:

(18) $$B_{D,1}(\theta )=a_{0}+a_{1}\cos\theta$$

(19) $$B_{D,2}(\theta )=a_{0}+a_{1}\cos\theta +a_{2}\cos^{2}\theta .$$

From the definition of the beam pattern and by using a Maclaurin series expansion, the following expression can be derived:

(20) $$\begin{array}{cc} B_{M}[h(\omega ),\theta ] & =\sum_{m=1}^{M}H_{m}(\omega )e^{j(m-1)\omega \tau_{0}}\\ & =\sum_{m=1}^{M}H_{m}(\omega )\sum_{n=0}^{\infty }\frac{1}{n!}{\left[j(m-1)\omega \tau_{0}\cos\theta \right]}^{n}\\ & =\sum_{n=0}^{\infty }\cos^{n}\theta [\frac{{\left(j\omega \tau_{0}\right)}^{n}}{n!}\sum_{m=1}^{M}{\left(m-1\right)}^{n}H_{m}(\omega )] \end{array}$$

where $\tau_{0}$ denotes the unit delay. Truncating the Maclaurin series to order *N* yields the following approximation for the beam pattern:

(21) $$B_{M,N}[h(\omega ),\theta ]=\sum_{n=0}^{N}\cos^{n}\theta [\frac{{\left(j\omega \tau_{0}\right)}^{n}}{n!}\sum_{m=1}^{M}{\left(m-1\right)}^{n}H_{m}(\omega )]$$

To achieve the desired beam pattern, an equation system is established by equating the corresponding coefficients of (17) and (21). Combined with the minimum norm method, the analytical expression for the system function h(ω) is then obtained.

### 3.3. Simulation Analysis

**Figure 11:**
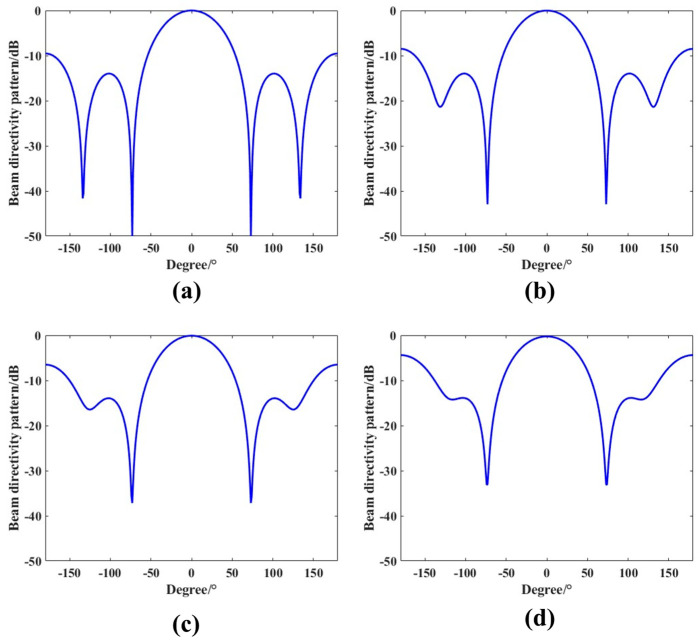
Simulated beam patterns of the second-order differential array at different frequencies. (**a**) 10 Hz. (**b**) 160 Hz. (**c**) 315 Hz. (**d**) 500 Hz.

To further evaluate the applicability of the DBF algorithm combined with the proposed MEMS hydrophone array, a simulation study was conducted. The array comprises six elements with an inter-element spacing of 0.25 m. Under the assumption of zero phase difference among the array element channels, the simulated signal bandwidth is set to 10–500 Hz. This configuration satisfies the condition that the inter-element spacing is much smaller than half the wavelength, with the sound speed taken as 1500 m/s. Figure 11 shows the output of the second-order differential beamformer applied to the MEMS hydrophone array.

**Figure 12:**
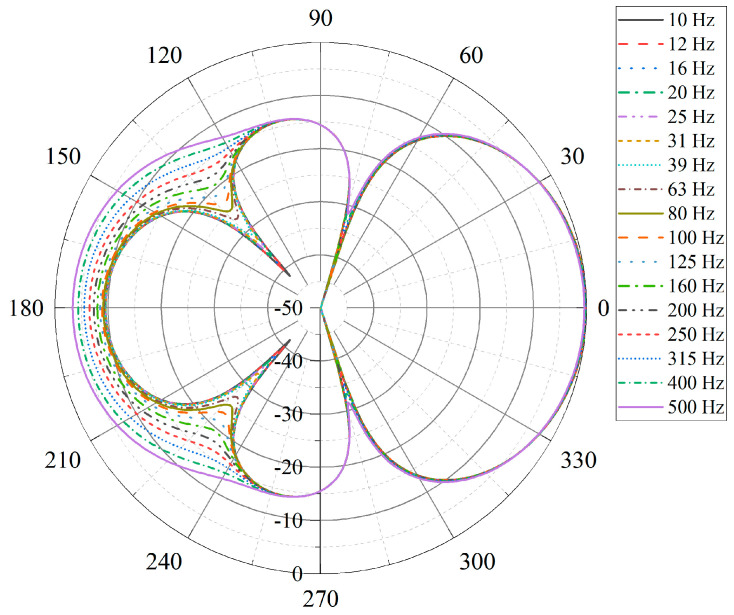
Beam patterns in polar coordinates for frequencies from 10 Hz to 500 Hz.

As shown in Figure 11, within the simulated frequency band, the beam patterns obtained from the second-order DBF exhibit consistent mainlobe and sidelobe characteristics across different frequencies. The corresponding polar-coordinate beam patterns are presented in Figure 12. These results confirm that the differential beam pattern is approximately frequency-invariant over the considered frequency range.

**Figure 13:**
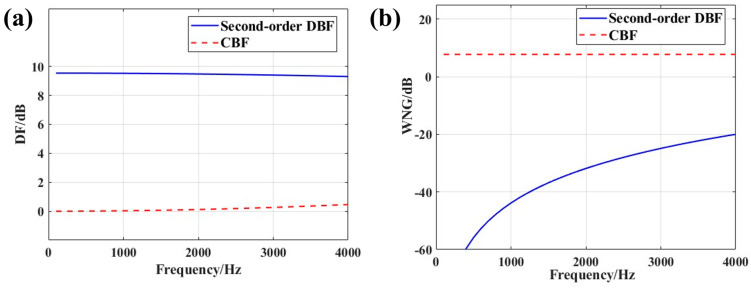
Performance comparison of CBF and second-order DBF. (**a**) DF. (**b**) WNG.

Under the same simulation conditions, a comparison of the directivity factor (DF) and white noise gain (WNG) between CBF and second-order DBF is presented in Figure 13.

Figure 13 compares the wideband performance of CBF and second-order DBF using the miniaturized MEMS array with ideal channel phase consistency. Figure 13a shows that the second-order DBF delivers a superior and stable directivity factor (7–10 dB) across the entire band, confirming its wideband constant-directivity advantage. Figure 13b reveals a key trade-off: CBF maintains a positive white noise gain across all frequencies, whereas the WNG of the second-order DBF deteriorates at higher frequencies, making it more susceptible to element noise and channel errors. Consequently, while second-order DBF is advantageous for low-frequency detection with the miniaturized MEMS hydrophone array, its practical deployment necessitates robust phase correction to maintain performance.

### 3.4. Sea Trial Data Validation

A phase-corrected miniaturized MEMS hydrophone array was tested for target detection performance in Qingdao coastal waters. A fixed sound source was positioned at a distance of 50 m from the array along its broadside direction, emitting a continuous sinusoidal signal with a center frequency of 20 Hz. The array position was defined within a Cartesian coordinate system, and the results were subsequently converted to target azimuth angles in relative coordinates. The processed results were compared with those obtained from conventional beamforming, where the array was aligned with the horizontal coordinate axis in the same coordinate system.

**Figure 14:**
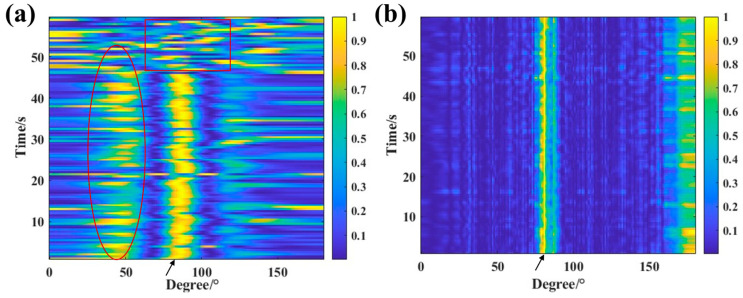
Target azimuth estimation results from sea trial data. (**a**) CBF. (**b**) DBF.

The data processing results are shown in Figure 14. As illustrated in Figure 14a, even when the element spacing is much smaller than half a wavelength, the CBF method still provides relatively stable azimuth estimates. However, as marked by the red ellipses in Figure 14a, the output suffers from severe sidelobe interference, which degrades the accuracy of target trajectory detection. In contrast, Figure 14b shows that, compared with conventional beamforming, the DBF method produces outputs where the interference levels are much lower than the output at the true target bearing (corresponding to the ellipses in Figure 14a), thus suppressing the interference and resulting in weak amplitude outputs. This allows DBF to provide more accurate target azimuth trajectory results. On the other hand, as indicated by the black arrows in both figures, the high-amplitude output in Figure 14a covers a wide range of azimuths, meaning the array output beam is broad, which degrades the accuracy of target azimuth estimation. In Figure 14b, the black arrows point to a narrower beamwidth and higher azimuth estimation accuracy, leading to a more precise representation of the target trajectory variation. Furthermore, during the latter part of the target motion, as marked by the red square in Figure 14a, it can be observed that Figure 14a fails to produce a stable target trajectory output, whereas Figure 14b still yields a stable target trajectory result.

## 4. Conclusions

This paper addressed the critical need for compact target detection systems on underwater mobile platforms by developing a detection system based on a miniaturized MEMS hydrophone array. Using AlN piezoelectric films and semiconductor fabrication technology, MEMS piezoelectric hydrophones were designed and fabricated. Acoustic pressure sensitivity and noise resolution tests demonstrated that the hydrophones achieve a sensitivity of −177.2 ± 1.5 dB (re: 1 V/µPa) across the 20 Hz–4 kHz frequency range and a noise resolution of 59.5 dB at 1 kHz, meeting the requirements of detection sonar systems. A six-element MEMS hydrophone array with 0.25 m spacing and 22 mm element diameter was constructed to enable target azimuth detection. To ensure algorithm robustness in practical applications, a phase self-correction method based on interleaved ADC control was proposed, enabling synchronous multi-channel signal acquisition and eliminating system-level phase errors. Through simulations and sea trials, DBF was compared with CBF using the miniaturized MEMS hydrophone array. The results demonstrate that DBF is better suited for low-frequency detection with the miniaturized MEMS hydrophone array. This work successfully achieves high-precision underwater target detection using a miniaturized MEMS hydrophone array, offering new insights and methodologies for sonar systems deployed on small underwater mobile platforms.

## Figures and Tables

**Table 1:** Characteristics of typical piezoelectric materials for MEMS hydrophone applications.

Piezoelectric Material	ZnO	AlN	PZT
Density (g/cm^3^)	5.61	3.3	7.8
Elastic modulus (GPa)	110–140	300–350	61
Hardness (GPa)	4–5	15	7–18
Piezoelectric constant *d*_31_ (Pc/N)	−5.74	−1.5	−40
Dielectric constant	8.66	8.5–10	380
Coefficient of thermal expansion (10^−6^/K)	4	5.2	1.75

**Table 2:** Finite element model parameters for modal analysis of the MEMS sensing chip.

Parameters	Values
Device silicon layer thickness	5 μm
Oxygen layer thickness	1 μm
Electrode thickness	0.3 μm
Piezoelectric layer thickness	1.8 μm

## Data Availability

The raw data supporting the conclusions of this article will be made available by the author on request.
